# Genotoxicity and gene expression modulation associated with the use of DDVP antiparasitic drugs in aquaculture on *Danio rerio*

**DOI:** 10.3389/fvets.2026.1832183

**Published:** 2026-05-12

**Authors:** Ad Viralkumar, Lukram Sushil Singh, Gowhar Iqbal, Vivek Shrivastava, Ridhdhisa R. Barad, Krinal Mori, Sujit Kumar

**Affiliations:** 1 College of Fisheries Science Kamdhenu University Himmatnagar, Gujrat, India; 2 ICAR-Central Institute of Fisheries Education, Panch Marg, Yari Road, Mumbai, India

**Keywords:** antiparasitic drug, aquaculture, *Danio rerio*, DDVP, gene expression, genotoxicity

## Abstract

**Introduction:**

Parasites are a significant threat to aquaculture, and the application of various antiparasitic chemicals by aquafarmers is a widely practiced strategy to control them. Few antiparasitic drugs are toxic to hosts at higher concentrations and may be genotoxic to them. Genotoxicity tests have been successfully used as indicators of the toxicity of several drugs and environmental pollutants, including heavy metals. The current study was aimed to assess the toxicity of antiparasitic chemicals or drugs at various levels, with special emphasis on genotoxicity in a Zebrafish model.

**Methods:**

Adult zebrafish were acclimatized under laboratory conditions and exposed to graded concentrations of dichlorvos to determine acute toxicity (LC25 and LC50) through a 96 h semi-static bioassay. Based on these values, fish were further subjected to sublethal and lethal concentrations for in vivo assessment at 24, 48, 72, and 96 h intervals. Genotoxicity was evaluated using micronucleus and alkaline comet assays on blood and gill tissues. Gene expression analysis of p53, Cyp1a, and Bcl-2 was performed in gill, liver, and kidney tissues using qRT-PCR after RNA isolation and cDNA synthesis. Data were statistically analyzed by one-way ANOVA followed by Tukey's *post-hoc* test at *p* < 0.05.

**Results:**

This study investigated the genotoxic potential of dichlorvos in zebrafish using micronucleus and comet assays, along with gene expression analysis of key biomarkers (p53, Cyp1a, and Bcl-2). The 96-hour LC50 and LC25 values of DDVP were determined as 10.6 ppm and 6.78 ppm, respectively. Based on sublethal toxicity, zebrafish (*Danio rerio*) were exposed to four concentrations (2.5, 5, 7.5, and 10 mg/L) for in vivo analysis. A significant, concentration- and time-dependent increase in micronucleus frequency was observed, with a 5.5-fold rise between the lowest and highest concentrations after 24 h (h). DNA damage, assessed through comet assay, was minimal at 24 h but increased non-linearly, reaching maximum levels at 96 h, particularly in the highest exposure group. Gene expression analysis revealed marked upregulation of p53, Cyp1a, and Bcl-2 in gill tissues, especially at higher concentrations. Bcl-2 showed the highest induction (19.1-fold), followed by p53 and Cyp1a, indicating activation of cellular stress, detoxification, and apoptosis-related pathways in response to DNA damage.

**Conclusion:**

Overall, dichlorvos exposure induced significant genotoxic effects in zebrafish in a dose- and duration dependent manner. The findings suggest that concentrations below LC25 may be relatively safer for therapeutic use. Further studies are required to check the efficacy of DDVP against different parasites to formulate more specific treatment strategies for sustainable aquaculture practices.

## Introduction

Aquaculture is the fastest-growing food-producing sector, contributing significantly to nutritional and livelihood security worldwide. Aquaculture offers advantages over agriculture in terms of its diverse nature, such as its ability to be practiced in all types of habitats, including freshwater, brackish water, and seawater. Aquaculture growth in India is exceptional and is expected to increase multi-fold in the coming years, mainly due to horizontal expansion, which is supported by the adoption of advanced aquaculture practices. Disease management is a major challenge for aqua-culturists, and farmers often face outbreaks in aquaculture systems. Parasitic diseases are highly prevalent problems that lead to morbidity and mortality and cause serious economic and ecological damage to aquaculture (1–3). Economic losses are pre-dominantly related to the mortality of cultured animals, expenditure on chemicals and drugs, reduced growth performance and increased feed conversion ratios. Some of the parasites that seriously impact aquaculture are Argulus, Lernaea, white spot, and Trichodynia (4). Various antiparasitic strategies are used in aquaculture, mainly involving chemicals and drugs. Different chemicals and drugs, such as diflubenzuron, teflubenzuron, emamectin benzoate, cypermethrin, deltamethrin, ivermectin, fenbendazole, chloramine-T, copper sulfate, malachite green, levamisole, metronidazole, potassium permanganate, and trichlorfon, are used in aquaculture to control parasites (5, 6, 30). These chemicals are used extensively, and higher doses are often used for the rapid removal of parasites. Many of these chemicals are toxic to fish. De Souse et al. (7) reported genotoxicity and carcinogenicity associated with ivermectin and amoxicillin in epithelial tumor test in Drosophila.

Genotoxicity tests have been successfully used as indicators of the toxicity of several drugs and environmental pollutants, including heavy metals. Fish are considered a good model for these types of analyses because of their ease of handling and monitoring under controlled conditions and low cost compared to other animal models. Genotoxicity studies analyze the damaging effects of a substance on the genetic material (DNA and RNA) of cells. Genotoxins are mutagens that can cause genotoxicity, harming DNA or chromosomal material and resulting in mutations (8). Zebrafish are widely accepted model organisms for studying drug toxicity (9). Analysis of the zebrafish genome has revealed over 70% of homologous genes with humans, and 82% of human genes associated with the disease have homologs in the zebrafish genome (10). Other advantages of zebrafish include optical transparency during early life stages, short generation time, continuous egg production, large number of offspring, rapid development, and cost-effectiveness (11). The current study aimed to assess the toxicity of antiparasitic chemicals or drugs at various levels, with special emphasis on genotoxicity in a Zebrafish model. Antiparasitic drugs were selected based on their availability and extent of application in aquaculture industry.

## Materials and method

### Ethical statement

All experimental protocols in the study were approved by the Institutional Animal Ethics Committee (IAEC) and Board of Studies (BoS) of the Postgraduate Institute of Fisheries Education and Research (PGIFER), Kamdhenu University Rajpur (Himmatnagar). India, with code no IAEC/BOS/PGIFER/012. All methods were carried out under relevant guidelines and regulations approved by the committee. The institute followed and approved all applicable international, national, and/or institutional guidelines for the care and use of zebrafish. Proper care was taken to minimize stress and suffering of the experimental animals.

### Location

The present study was conducted at the Central Biotechnology Laboratory, Postgraduate Institute of Fisheries Education and Research (PGIFER), Kamdhenu University Rajpur (Himmatnagar). Zebrafish toxicity assessment was performed at the Zebrafish Research Laboratory, PGIFER, Kamdhenu University, Rajpur (Nava) Himmatnagar.

### Experimental fish

Healthy adult Zebrafish (Bloch, Family Cyprinidae and Order Cypriniform's) were procured from the local outlets. The average weight of the fish was 0.48 ± 0.06 g, and the average length was 3.7 ± 1.8 cm. The fish were given prophylactic treatment following the standard protocol of bath treatment in a 0.05% potassium permanganate solution before placing them in an acclimatization tank. Fish was reared for 1 month under laboratory conditions for proper acclimatization under the optimal conditions as per the suggestion of Bennett and Dooley (32) before Dichlorvos exposure. The fish were fed a floating feed. Fecal matter and unutilized feed were siphoned off daily, along with partial water replacement, to maintain optimum water quality.

### Experimental design

The experiment was conducted in triplicate for each treatment, with 15 healthy adult zebrafish (active and no disease symptoms) in each replicate group. Therefore, the total number of fish used in the experimental groups was 270 for 96 h. Technical-grade dichlorvos (effective concentration 76%) under the trade name NUVAN (manufactured by Insecticides India Ltd.) was purchased from the market. The concentration of DDVP was diluted to the required concentrations in distilled water for the acute toxicity assessment. The sample size was determined based on standard experimental design and previous similar toxicological studies to ensure reliability and statistical validity of the results. Each treatment was conducted in triplicate with 15 fish per replicate to minimize variability and allow proper statistical analysis.

### Determination of sub-lethal concentrations

The acute toxicity bioassay of dichlorvos was performed in a semi-static culture system in a glass aquarium (10 L capacity) with the exchange of test water was exchanged every alternate day for the determination of both LC_25_-96 h and LC_50_-96 h values. A continuous aeration facility was provided to maintain optimum dissolved-oxygen levels in the test water. The standard procedure for acute bioassays was followed according to the APHA (33) guidelines. All three experiments were conducted with varying concentrations of dichlorvos, ranging from 0.5 to 25 mg/L, for the acute toxicity bioassay. The experiment was performed in triplicate for each treatment, with 15 healthy (active and no disease symptoms) adult zebrafish in each replicate group. The fish were randomly exposed to each of the five dichlorvos target concentration levels in experiment 1 (0, 0.5, 1, 1.5, 2, and 2.5 mg/L), experiment 2 (0, 2.5, 5, 7.5, 10, and 12.5 mg/L), and experiment 3 (0, 5, 10, 15, 20, and 25 mg/L). The LC_50_ value of dichlorvos was determined using probit analysis with spssv24. Based on the LC_50_ and LC_25_ values, *in vivo* experiments were conducted to assess genotoxicity and gene expression.

### *In vivo* exposure experiment

The findings of the acute toxicity bioassay were used to design *in vivo* exposure experiments for a detailed investigation of genotoxicity and gene expression. A total of four test concentrations of dichlorvos were selected as concentration I (1/4th of LC_50_), concentration II (1/2 of LC_50_), concentration III (3/4th of LC_50_) and concentration IV (LC_50_) for the study. Chemical exposure was continued for 96 h (4 days), and tissue and blood sampling was performed at intervals of 24, 48, 72, and 96 h at the rate of six fish per treatment at every time point. The negative control was maintained in the same water without the addition of dichlorvos. Blood and gill samples were collected and processed on the same day for the micronucleus test and comet assay, and another tissue sample was collected using a standard protocol for gene-expression analysis. The physicochemical properties of the test water, including temperature, pH, total conductivity, dissolved oxygen, and total hardness, were assessed using standard methods (33).

### Micronucleus assay

The zebrafish were anesthetized in chilled water, and their body surfaces were carefully wiped with tissue paper. Blood from the fishtail was directly collected by cutting the caudal and anal fins on a glass slide containing one drop of 2% ethylenediaminetetraacetic acid (EDTA; anticoagulant). Immediately after blood collection, a thin smear was prepared using a glass slide. The slides were air-dried for 10 min and stained with field staining (Himedia kit) after fixation in absolute methanol. The kit protocol was followed with minor modifications, as described below. The stained slides were air-dried and observed under an oil-immersion objective at 100x magnification. The parameters used to identify micronuclei included no connection with the main nucleus, the same color and intensity as the main nucleus, and an area smaller than one-third of the main nucleus. The total number of cells and the number of cells containing micronuclei were counted for each treatment, and the frequency of micronucleus was calculated using the following formula:


$$\begin{array}{c} \text{MN\%}=(\text{Number of cells containing micronucleus}/\text{total}\\ \text{number of cells counted})\text{ x }100 \end{array}$$

### Alkaline single-cell gel electrophoresis (SCGE)

Alkaline single-cell gel electrophoresis (SCGE)/ comet assay was performed as a three-layer procedure (34) with slight modifications using conventional microscopic slides. First, the slides were cleaned with 100% ethanol and flame-dried. Gill and blood were used for comet assays. The glass slides were first dipped in methanol, wiped with tissue paper to remove any residual oil and dirt, and labeled. The base layer coat was prepared using a 1% agarose solution (1x PBS) on a frosted slide. The slide-in slide tray was placed on a flat surface for air drying. The gill was dissected from the zebrafish and placed (50 mg) in 500 μl PBS in a microcentrifuge tube. Gill tissue was homogenized using scissors and a tissue homogenizer. The supernatant was transferred to another tube and kept on ice to reduce the enzymatic activity. This cell suspension (20 μl) was mixed with 80 μl LMPA (low low-melting-point agarose LMPA (Lonza) for further use. Blood (25 μl) collected from the fish was mixed with 225 μl ice-cold 1 × PBS solution in a microcentrifuge tube and mixed properly. The cell suspension (20 μl) was mixed with 80 μl LMPA [0.5% in phosphate-buffered saline (PBS)]. A total of 100 μl of this suspension was placed over the previously prepared slide (1st layer coated), and a glass coverslip was placed over it, carefully avoiding the formation of air bubbles. The slides were kept over ice packs/ice cubes for rapid solidification or were left to air dry. After drying, the coverslip was moved, and 100 μl of low-melting agarose (0.5% in PBS) was poured (3rd layer) over the second layer, and the coverslip was again placed and left to dry. The coverslip was removed again after drying, and the slide was placed in a Coplin jar containing 45 ml of chilled lysis solution along with 4.5 ml of 10% DMSO and 0.5 ml of 1% Triton-X 100 and left overnight for cell lysis. All processes were performed under a dim light. After lysis, the slides were electrophoresed. The pH of the electrophoresis buffer must be checked before use and should be greater than 13. The slide was kept in electrophoresis buffer for 20 min, and a 20-min electrophoretic run was performed while maintaining a current of 300 mA and voltage of 18V. The unit was switched off after 20-min runs, and the slides were removed from the electrophoresis buffer and drained properly before placing them in the neutralization buffer for 5 min. The above two steps (electrophoresis and neutralization) were repeated twice. After the above steps, the slides were placed in a Coplin jar containing absolute ethanol for dehydration. After 10 min, the slides were removed and stained with an ethidium bromide solution. The slides were then used for comet-image analysis. A minimum of two slides per sample and 25 comets per slide were observed using comet image analysis software after capturing images using a fluorescent microscope with specific filters. The percentage of tail DNA was estimated using this software. One-way analysis of variance was employed to compare the mean differences in % tail DNA between tissues within concentrations, between concentrations within tissues, and between durations within concentrations and tissues.

### Collection of tissues for gene expression study

The collected tissue samples (gills, liver, and kidney) from each treatment were immediately stored in individual tubes in RNA. Later, after 24 and 96 h. Three fish from each of the three replicates of every treatment were sacrificed at every sampling time point and stored in RNA Later. The dissected tissue samples were archived at −20 °C for a few days before RNA isolation and further analysis.

### RNA isolation and cDNA preparation

RNA was isolated from the tissue samples using the TRIzol reagent. RNA samples were visualized on a 1% agarose gel. All preparations for agarose gel electrophoresis were performed using DEPC-treated double-distilled water (DDW). The casting tray, comb, and buffer tank were washed with DEPC-treated water and dried. The gel was visualized using a gel documentation system. The quality and concentration were also analyzed using a micro-spectrophotometer (Qiaxpert). Complementary DNA was prepared from total RNA isolated from various tissues. First-strand cDNA synthesis was performed using 1 μg of DNase-treated total RNA template. cDNA was synthesized using the Verso cDNA synthesis kit (Thermo Scientific, USA) according to the manufacturer protocol. Briefly, 1 μg of total RNA was mixed with 0.5 μg of oligo (dT) primer, 4 μl 5x cDNA synthesis buffer, 2 μl dNTP mix (5 mM each), 1 μl RT enhancer, 1 μl verso enzyme mix, and nuclease-free water to a final volume of 20 μl. The reaction mixture was placed at 42 °C for 30 min and inactivated at 95 °C for 2 min. and immediately stored on ice for subsequent analysis. The synthesized first-strand cDNA was directly used in PCR to amplify the target cDNA using specific primers, as described by Sambrook and Russell (12). Gradient PCR was performed to optimize the annealing temperature for different target genes. The annealing temperature and PCR cycling conditions varied for each set of primers, which were standardized by performing gradient and touchdown PCR.

### Real-time PCR analysis

Real-time PCR was performed on a Rotor-Gene Q (Qiagen) thermocycler using 2x MaximaTM SYBR Green qmaster mix (2x). The reactions were set in 12.5 μl of total volume using 1μl of cDNA reverse transcribed from 1μg of total RNA, forward and reverse primers (0.5 pMol each), 2x SYBR Green master mix, and nuclease-free water to make up the final volume. The PCR cycle consisted of an initial denaturation step at 94 °C for 5 min, followed by an amplification step (40 cycles of 94 °C for 30 sec/60 °C for 20 sec/72 °C for 30s), a single melting curve step of 95 °C for 5 sec/65 °C for 1 min/97 °C, and finally a cooling step of 40 °C for 10s. The raw Ct data from the real-time experiments were converted into fold-change values using the 2^−ΔΔCt^ method (35). The fold change data were further analyzed statistically using ANOVA, followed by Duncan's test using the SPSS v24 software. Primers used for gene expression study are listed in the Table 1.

**Table 1:** List of primers used for gene expression analysis of target genes (*p53, Cyp1a*, and *Bcl-2*) along with housekeeping genes used for normalization.

Sr. no	Gene name	Sequence	Annealing temperature
1	Cyp*P450*	F: TCGCTCCGGGTTATTAAATCAGC R: CGCATGAGCAGATACACCAAAC	60 °C
2	*ZF_Bcl-2*	F: AGGAAAAGGAGGTTGGGATG R: TGTTAGGTATGAAAACGGGTGGA	60 °C
3	*ZF_ β-ACTIN*	F: CGAGCAGGAGATGGGAACC R: CAACGGAAACGCTCATTGC	60 °C
4	*ZF_P53*	F: TTGTCCCATATGAAGCACCA R: TTTCCTGTCTCTGCCTGGAC	60 °C

### Statistical analyses

Data were analyzed using one-way analysis of variance (ANOVA), followed by Tukey's *post hoc* test, using IBM SPSS Statistics (IBM Corp., USA). Results are expressed as mean ± standard deviation (SD). Statistical significance was set at *p* < 0.05 (95% confidence level).

## Results

### Physico-chemical properties of the test water

The physicochemical parameters of water used for both sub-lethal concentration and *in vivo* studies were maintained within the optimum range and are presented in Table 2. The water temperature varied from 26.3 to 28.1 °C, and pH values ranged from 7.2 to 8.278. The dissolved oxygen concentration was maintained above 6 ppm throughout the experiment by employing continuous aeration of the water. The conductivity of the water ranged from 400 to−546 μm/cm. The total dissolved solids (TDS) ranged from 210 to 273 mg/L, and total alkalinity ranged from 250 to 300 mg/L as CaCO_3_. Other parameters, such as ammonia and nitrate, were maintained within the optimal range through frequent water exchange (on a daily basis).

**Table 2:** The physicochemical parameters of water used for both sub-lethal concentration and *in vivo* studies.

Parameters	Range
Temperature	26.3–28.1 °C
pH	7.2–8.278
Water conductivity	400–546 μm/cm^−1^
Total hardness	250–300 mg/L
TDS	210–273 mg/L
Salinity	0 **±** 0.20 ppt
Resistivity	1.83 KΩcm

### Sub-lethal concentration of DDVP

**Figure 1:**
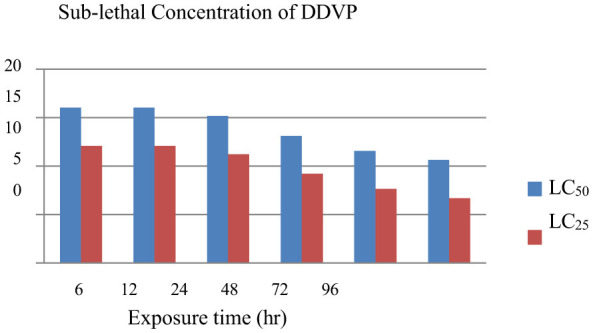
Sub-lethal toxicity concentration of dichlorvos (DDVP) in zebrafish at different exposure durations.

Three trials with varying concentration ranges (0.5–25 mg/L) were conducted to determine the sub-lethal concentration of dichlorvos. The LC_25_ and LC_50_ values were calculated for all time points (6, 12, 24, 48, 72, and 96 h), following the probit analysis mentioned in the methodology and presented in Table 3. The LC_50_ and LC_25_ values after 96 h, exposure was 10.612 and 6.696, respectively. The values at different time points are also compared graphically, as shown in Figure 1. DDVP is used as an antiparasitic drug; therefore, LC_25_ values were estimated to determine the safe treatment concentration.

**Table 3:** LC_50_ and LC_25_ values of dichlorvos (DDVP) at different exposure durations (6, 12, 24, 48, 72, and 96 h) in zebrafish.

Experiment of hour	LC_50_	LC_25_
6	16.008	12.092
12	16.008	12.092
24	15.143	11.227
48	13.122	9.206
72	11.564	7.648
96	10.612	6.696

### *In vivo* exposure experiment

Based on the findings of the sublethal toxicity results, four test concentrations of dichlorvos were selected for the *in vivo* study: concentration I (2.5 mg/L), concentration II (5 mg/L), concentration III (7.5 mg/L), and concentration IV (10 mg/L). The experiment was performed as explained in the Materials and Methods section for 96 h. The sample was analyzed using the micronucleus test, comet assay, and important gene expression studies. The following subsections emphasize the findings of the MNT and comet assay.

### Induction of micronuclei's test

**Figure 2:**
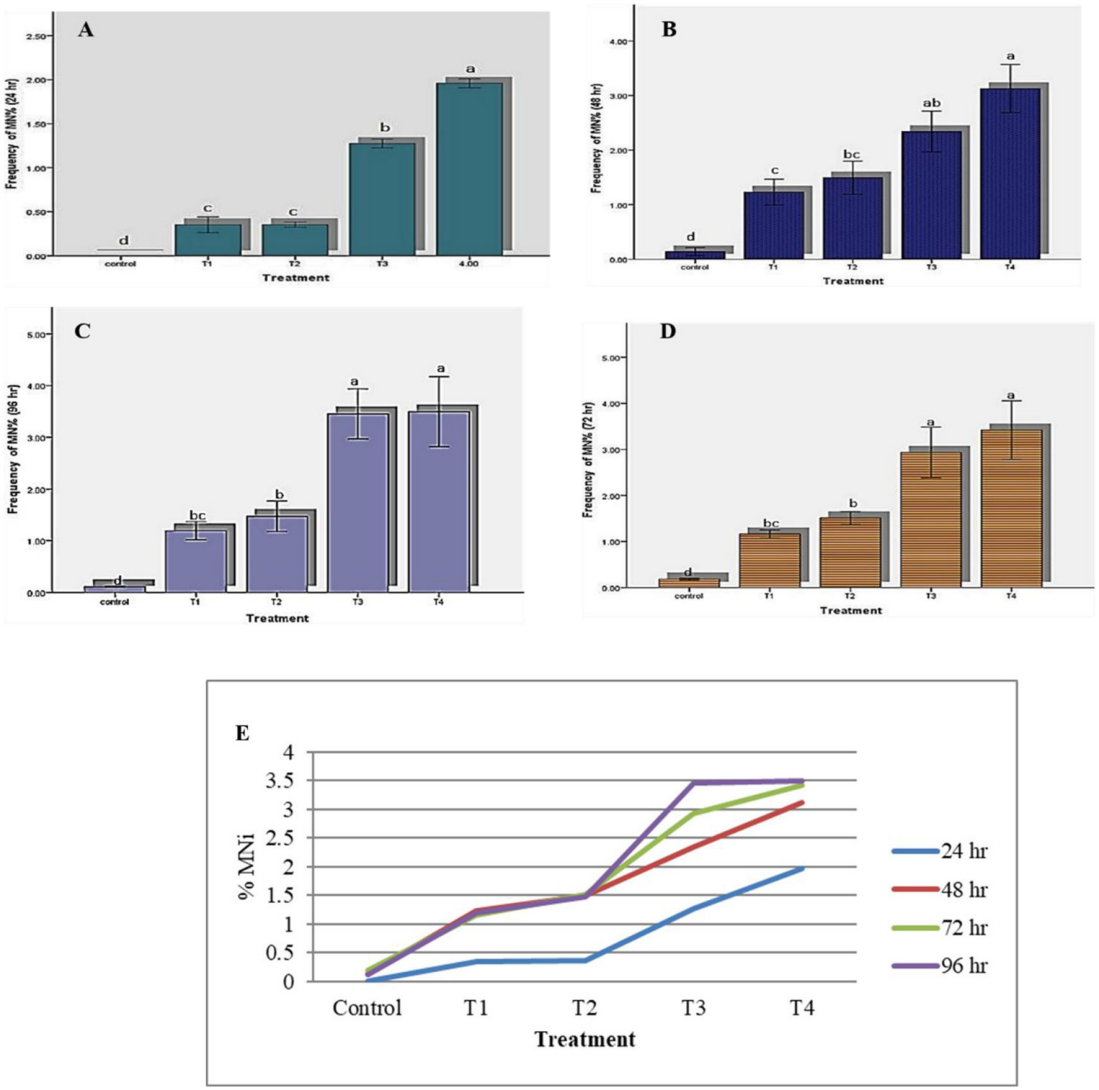
Percentage of MNi frequency at different time intervals. **(A)** 24 h; **(B)** 48 h; **(C)** 72 h; **(D)** 96 h; **(E)** Combined percentage of MNi Frequency. Data is presented as means ± SD of three replicates. Different superscript letters on different bars indicate a significant difference (*p* < 0.05) between different concentration within durations.

**Figure 3:**
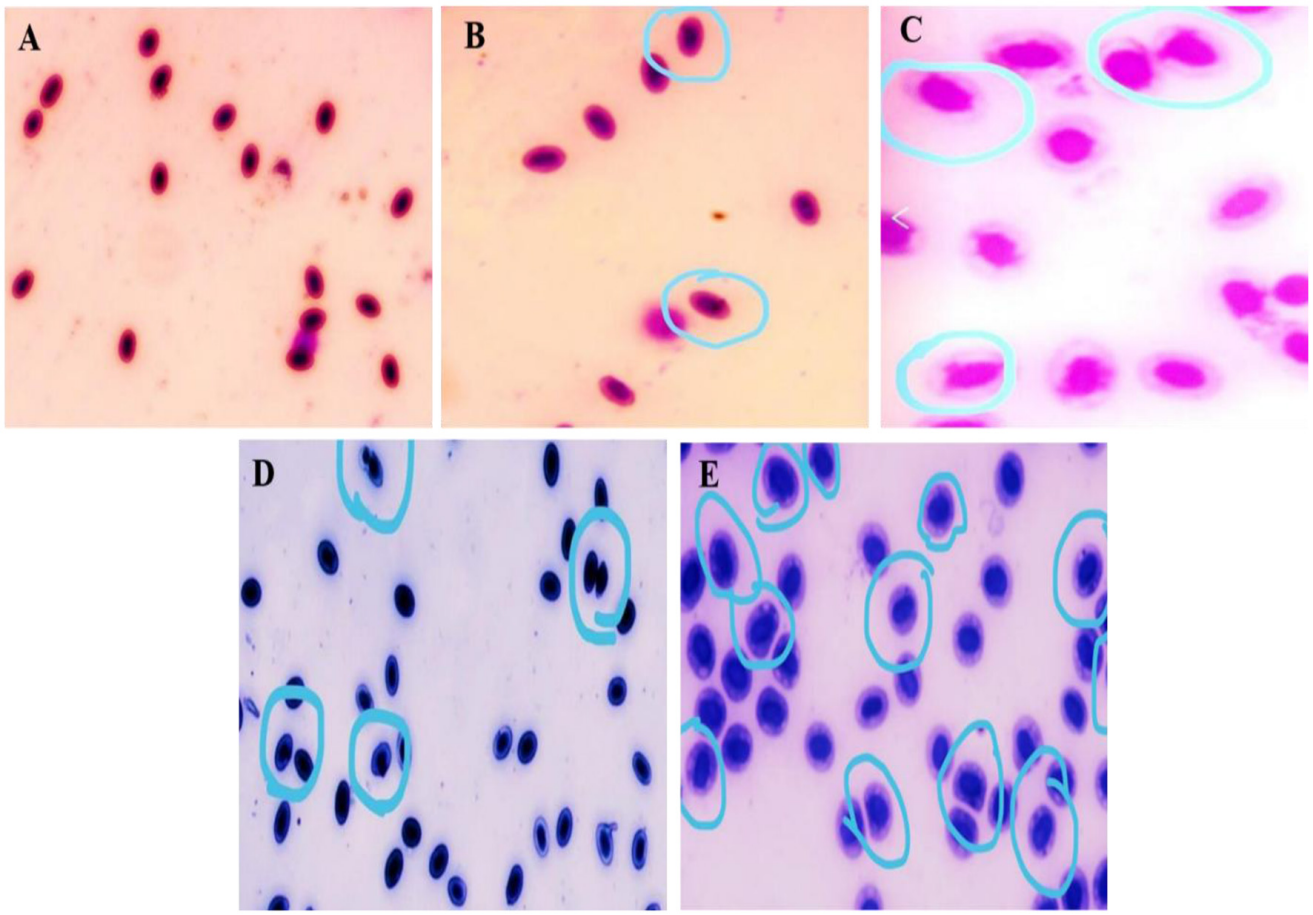
MNT image of different treatment. **(A)** Control (No micronuclei); **(B–E)** Treatment 1–4; Encircle indicates presence of micronuclei in treatments groups.

The results of the micronucleus test were analyzed for different concentrations at different time points to examine the effects of concentration and time of exposure on DNA damage, employing the development of micronuclei and associated nuclear deformities. The results indicated a concentration-and exposure time-dependent increase in micronucleus frequency compared to that in the control (Table 4). A 5.5-fold increase in micronucleus frequency was observed between T1 (2.5 ppm) and T4 (10 ppm) at 24 h. intervals, whereas the increase between T1 and T3 was 3.6-fold. A similar pattern was observed at all time points between the higher and lower DDVP concentrations. The MN frequency for a particular treatment showed a several-fold increase from 24 to 96 h. Furthermore, a significant (*P* < 0.05) effect of duration on the induction of MNi was observed for all concentrations of DDVP. The lowest concentration of DDVP treatment (2.5 ppm) induced MNi frequency of 0.3519% in erythrocytes in 24 h, which significantly increased to 1.1939% after 96 h of exposure (Figure 2). A similar trend was observed for concentration II, in where the MNi frequency of 0.3534% in 24 h increased to 1.4717% after 96 h. However, at the highest concentration, the increase in MN induction was 34 comparatively lower (1.78-fold for T4) (Figure 3).

**Table 4:** Frequency of MNi induced by DDVP in erythrocytes of *Danio rerio* at different concentrations and exposure intervals.

Exposure time (h)	Dosage	Total cells scored	Frequency of MN% ±SE
24	Control	892	0.0000 ± 0.000^d^
	T1	569	0.3519 ± 0.08897^c^
	T2	741	0.3534 ± 0.0289^c^
	T3	658	1.275 ± 0.0506^b^
	T4	734	1.9594 ± 0.05204^a^
48	Control	430	0.1404 ± 0.07134^d^
	T1	639	1.2305 ± 0.23586^c^
	T2	496	1.4937 ± 0.30261^bc^
	T3	803	2.3408 ± 0.37272^ab^
	T4	960	3.1259 ± 0.44107^a^
72	Control	447	0.184 ± 0.01984^d^
	T1	605	1.1624 ± 0.08803^bc^
	T2	398	1.5102 ± 0.13869^b^
	T3	457	2.9326 ± 0.55162^a^
	T4	648	3.4177 ± 0.63668^a^
96	Control	763	0.1128 ± 0.00913^d^
	T1	589	1.1939 ± 0.17692^bc^
	T2	583	1.4717 ± 0.29408^b^
	T3	1,036	3.4523 ± 0.4839^a^
	T4	1,213	3.4942 ± 0.67645^a^

Values with numeric superscripts differ significantly (*P* < 0.05) between durations within concentrations. Data is presented as means ± SD of three replicates. Different superscript letters on different bars indicate a significant difference (*p* < 0.05) between different concentration within durations.

### Comet assay (DNA damage)

**Figure 4:**
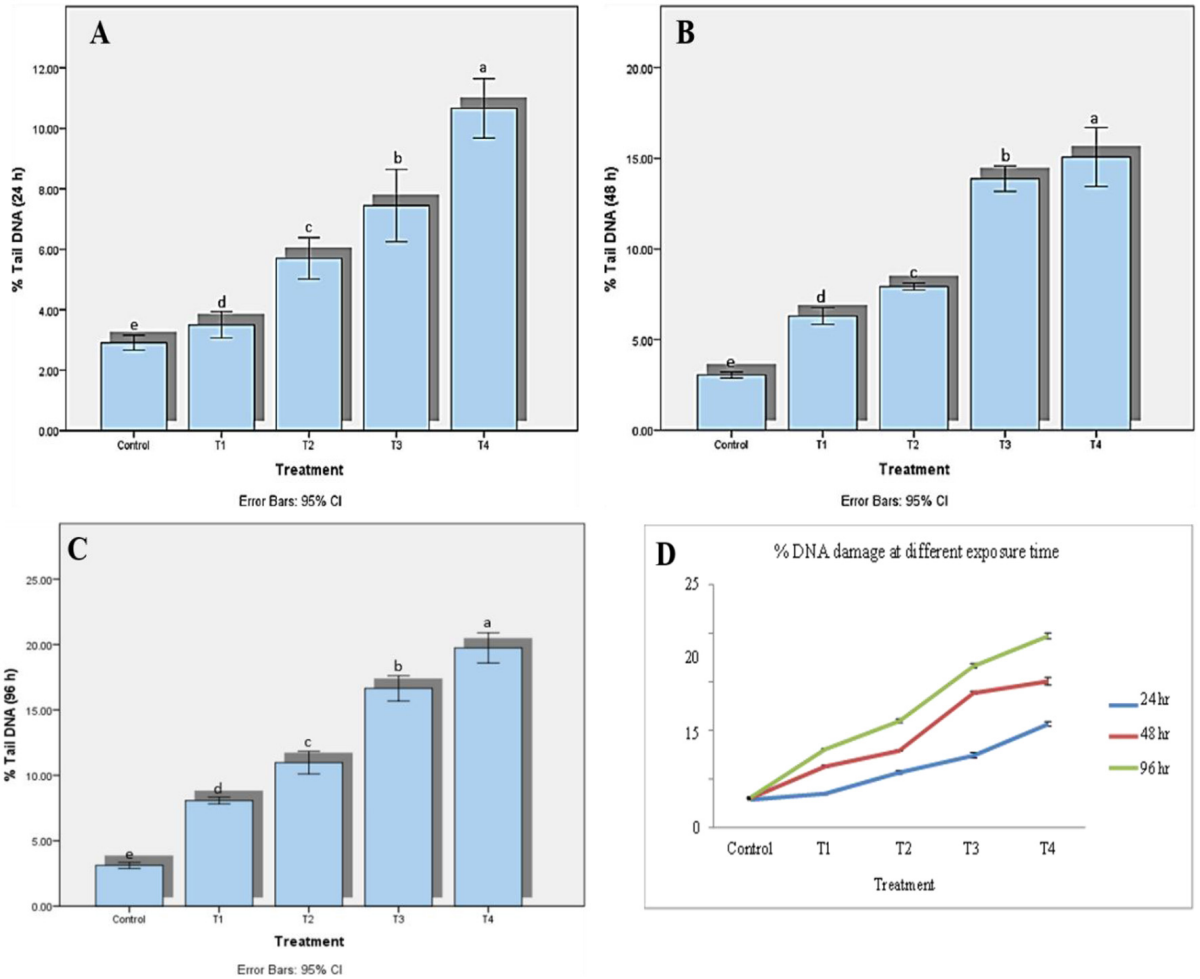
Effects of different dichlorvos (DDVP) treatments on percentage of tail DNA in zebrafish at various exposure durations. **(A)** 24 h, **(B)** 48 h **(C)** 96 h, and **(D)** combined effect of DNA damage across all exposure times. A significant increase in DNA damage was observed with increasing concentration and exposure duration, with maximum damage observed at 96 h. Data are presented as mean ± SD (*n* = 3). Different superscript letters on different bars indicate a significant difference (*p* < 0.05, Tukey's HSD test) at different exposure times.

**Figure 5:**
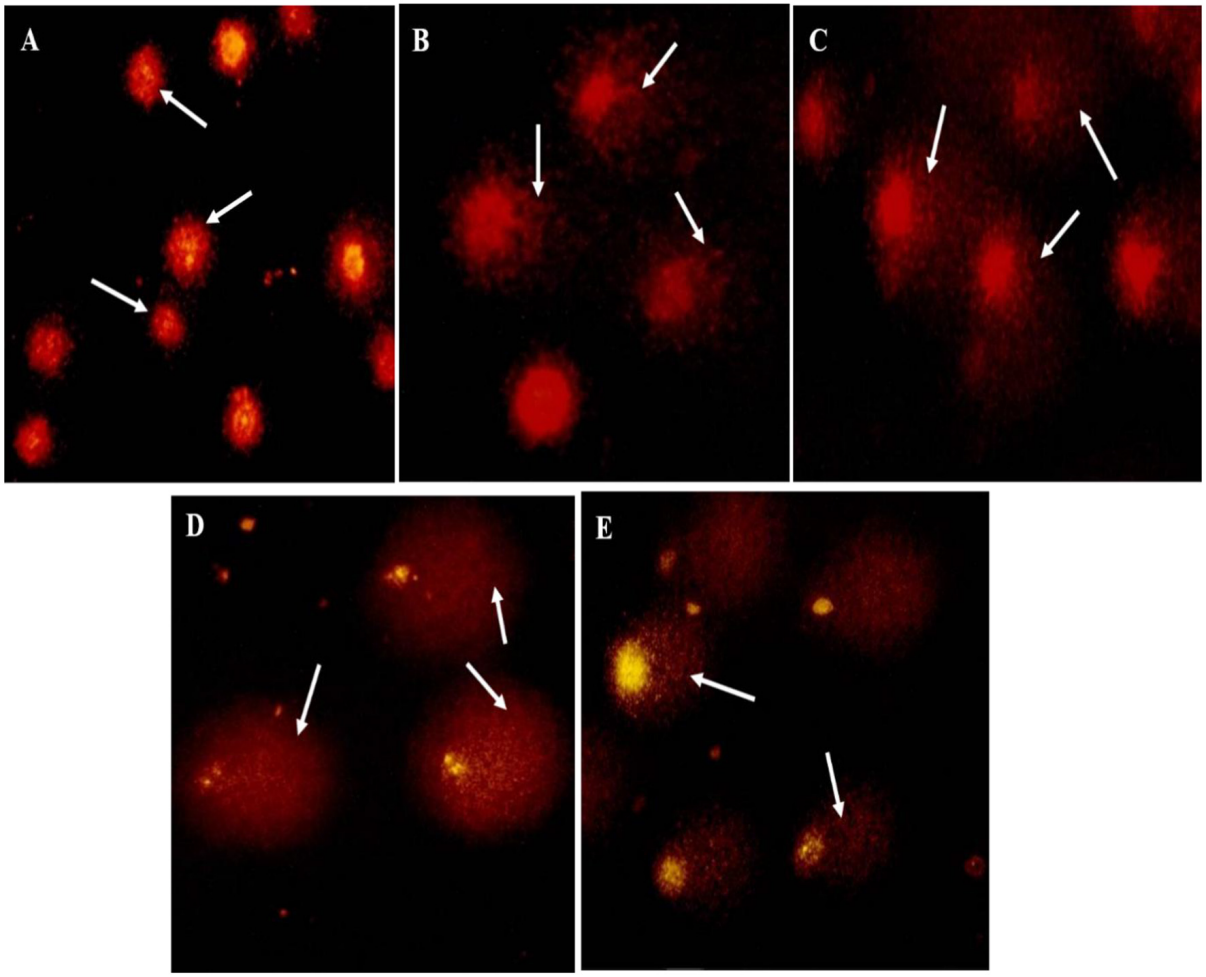
Detection of DNA damage in zebrafish using the comet assay under different dichlorvos (DDVP) treatments across various exposure durations. **(B–E)** show treated groups, where DNA damage is indicated by comet tail formation (marked by arrows), while control **(A)** shows intact nuclei with no observable DNA damage.

Zebrafish treated with DDVP exhibited significantly higher DNA damage (*P* < 0.05) in their tissues at higher concentrations than at lower concentrations and controls. A significant effect of exposure duration and concentration (*P* < 0.05) was observed in the zebrafish gills exposed to DDVP. The lowest DNA damage was observed at 24 h, and there was a gradual non-linear increase in DNA damage in the gill tissue as the experiment progressed. The highest DNA damage was observed after 96 h for all the treatments. The study also showed a significant effect (*P* < 0.05) of DDVP concentration on the induction of DNA damage in lymphocytes. In general, DNA damage was found to be concentration-dependent, with the highest DNA damage at concentration IV, followed by concentrations III, II, and I (Table 5 and Figure 4). DNA damage at different treatment across different time interval is shown in Figure 5.

**Table 5:** DNA damage (% tail DNA) in zebrafish exposed to different concentrations of dichlorvos (DDVP).

Exposure time (h)	Dosage	Mean ±SE (% tail DNA)
24	Control	2.9067 ± 0.05783^e^
	T1	3.5 ± 0.10116^d^
	T2	5.6967 ± 0.1593^c^
	T3	7.4433 ± 0.27841^b^
	T4	10.6633 ± 0.22806^a^
48	Control	3.0467 ± 0.03756^e^
	T1	6.3033 ± 0.10713^d^
	T2	7.9267 ± 0.0441^c^
	T3	13.8733 ± 0.16169^b^
	T4	15.07 ± 0.37754^a^
96	Control	3.1167 ± 0.05364^e^
	T1	8.0733 ±0.0.05925^d^
	T2	10.9767 ± 0.20103^c^
	T3	16.6467 ± 0.22578^b^
	T4	19.74 ± 0.26764^a^

A significant increase (*P* < 0.05) in DNA damage was observed with increasing concentration, with the highest damage recorded in concentration IV, followed by III, II, I, and control.

Values represent mean differences among treatments, with different superscript letters indicating significant differences (*P* < 0.05) at different exposure times.

### Concentration and purity of total RNA

**Figure 6:**
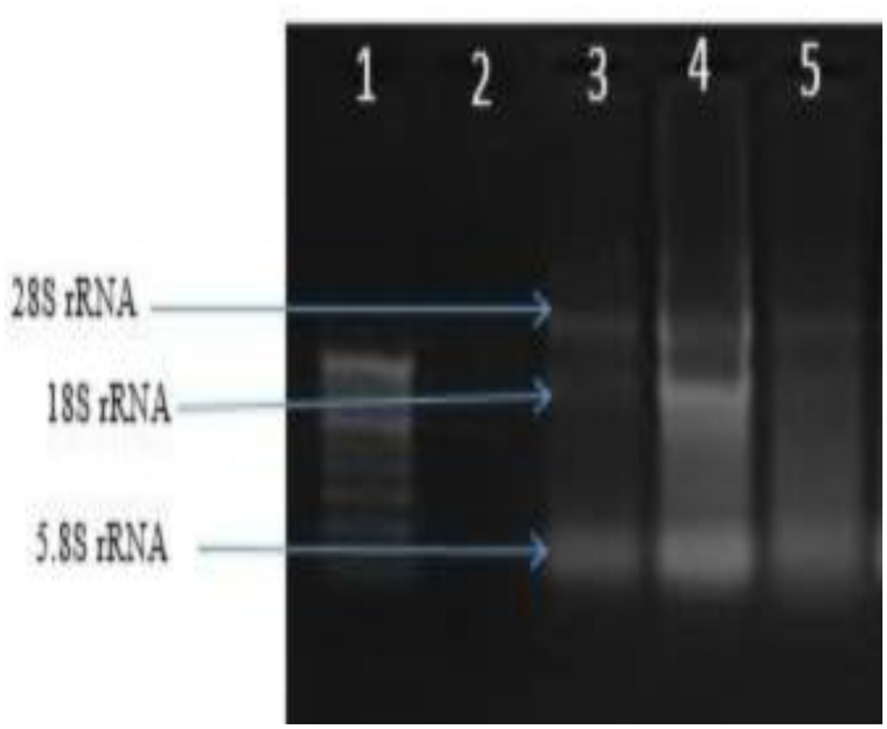
RNA isolation; Lane 1: ladder (100 bp); Lanes 3 to 5: RNA isolated from gill sample.

Total RNA was extracted from the gill tissues of zebrafish exposed to various concentrations of DDVP using the TRIzol method (Figure 6). It was quantified using a QIAxpert spectrophotometer and was found to be in the range of 450–1,000 ng/μl. Total RNA was subjected to cDNA synthesis after DNase I treatment. The cDNA quality was checked by PCR amplification of the β-actin gene (housekeeping gene).

### Optimization of primers using gradient PCR

**Figure 7:**
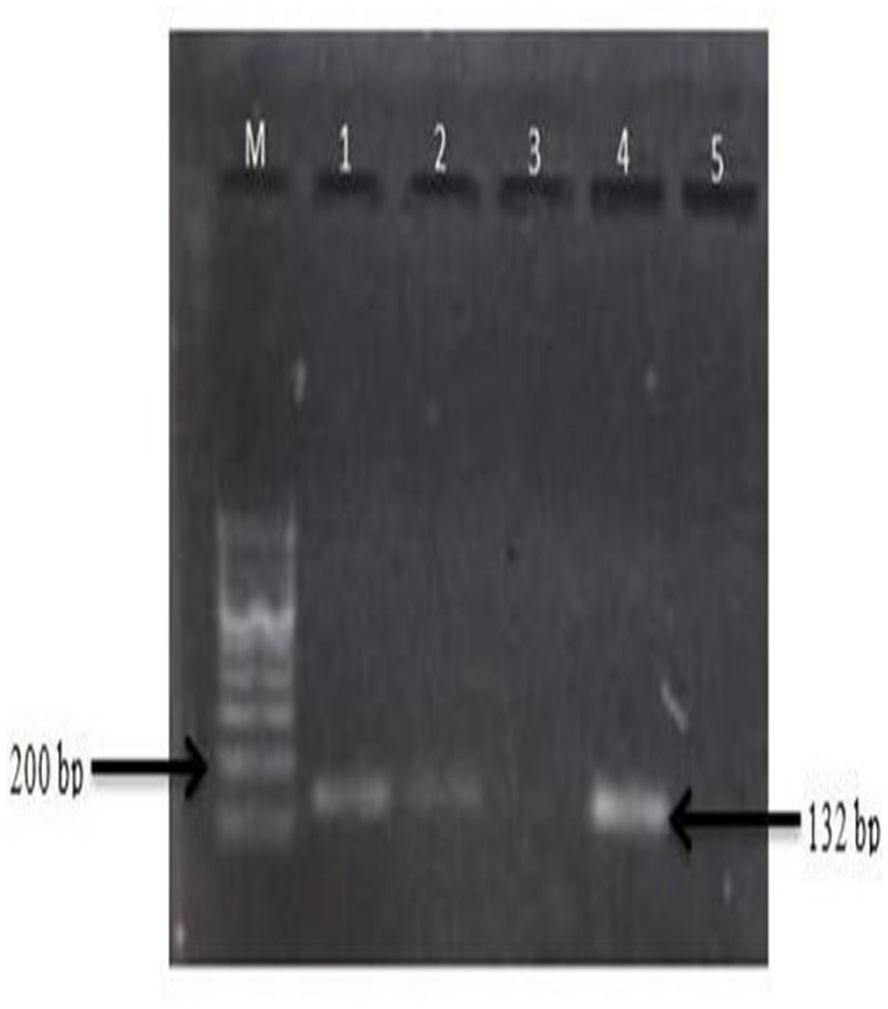
Gradient PCR of *p53* (1-55 °C, 2-57 °C, 3-58 °C, 4-60 °C, 5-62 °C) M: DNA ladder (100 bp); lanes: 1 to 5 (different annealing temperature).

Gradient PCR was performed in the temperature range of 55–62 °C to determine the optimum annealing temperature, and the results are shown in Figure 7. The image clearly shows the best amplification with minimum dimer at 60 °C for all three genes (*p53, Cyp1a*, and *Bcl-2*). The PCR composition is presented in (Table 6). Expression studies were performed after determining the optimum annealing temperature.

**Table 6:** PCR reaction composition used for gradient PCR.

cDNA sample	0.5 μl
Buffer (10x)	2.5 μl
Primer (F) 10 Pmol/μl	0.5 μl
Primer (R) 10 Pmol/μl	0.5 μl
Tag polymerase (five unit/L)	0.15 μl
DNTP Mix (2 mm each)	0.5 μl
Nuclease-free water (to make final reaction volume 25 μl)	20.35 μl

### Expression studies of genotoxicity related genes

**Figure 8:**
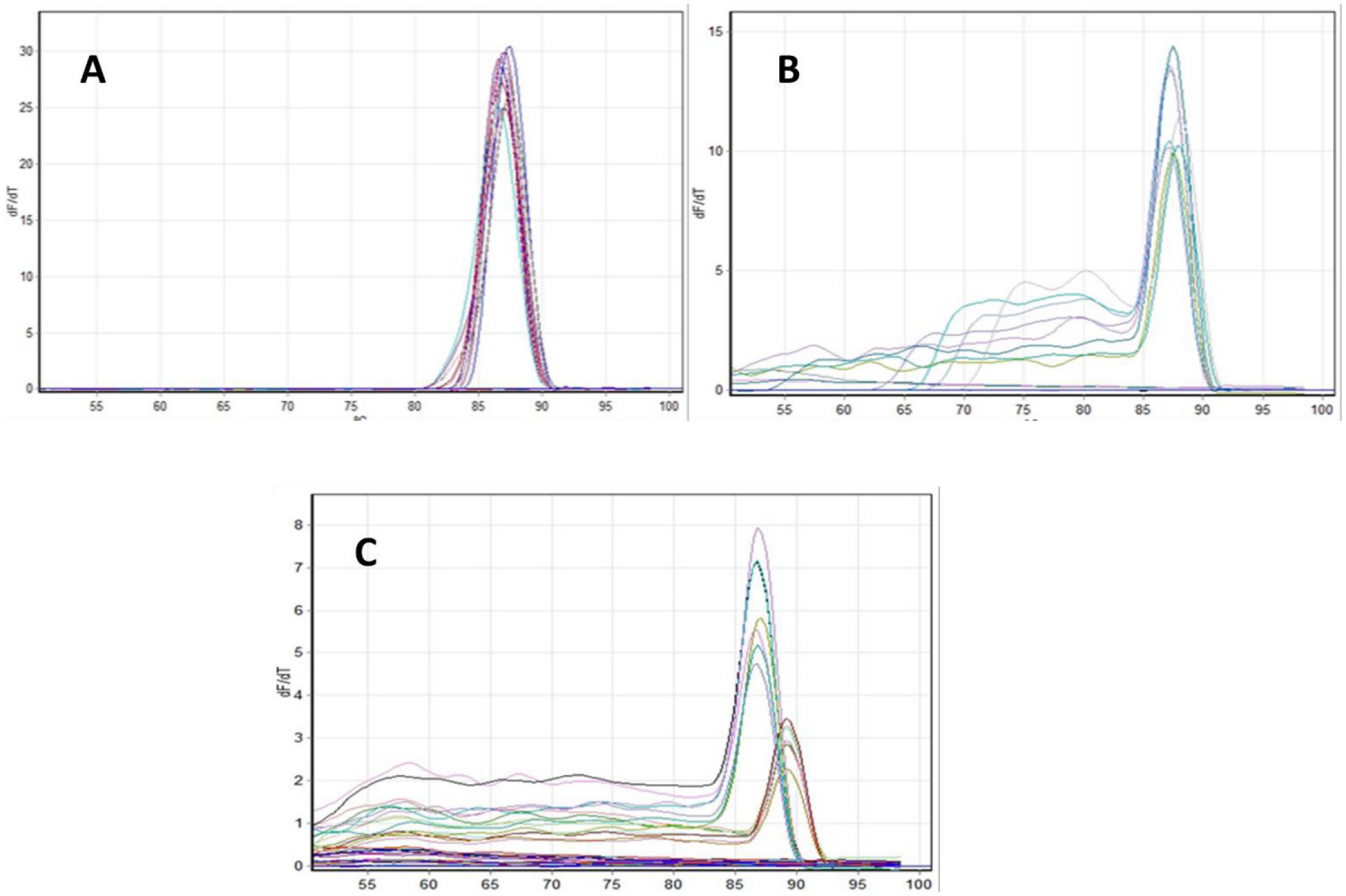
Melting curve of β-actin **(A)**; *Bcl-2* **(B)**, *p53* and *Cyp1a* **(C)** gene melting curve.

Expression profiling of genotoxicity related genes, namely *p53, Cyp1a* and *Bcl-2* were performed by real-time PCR. The expression of housekeeping genes namely β-actin, EF1α and GAPDH were also analyzed to identify the most stable internal control gene. The expression of β-actin was found to be the most stable among three and finally used as an internal control for gene expression study. Expression analysis was carried out in gill tissues of all treatments post 96 h exposure. Melting curve/peak analysis was done for all the genes after each qRT-PCR, which showed a specific product amplification by each primer pair as shown in Figure 8. The Ct value was used for data analysis as per the protocol mentioned in materials and method. One-way anova was performed using the software SPSS v24.

### *p53* gene expression

**Figure 9:**
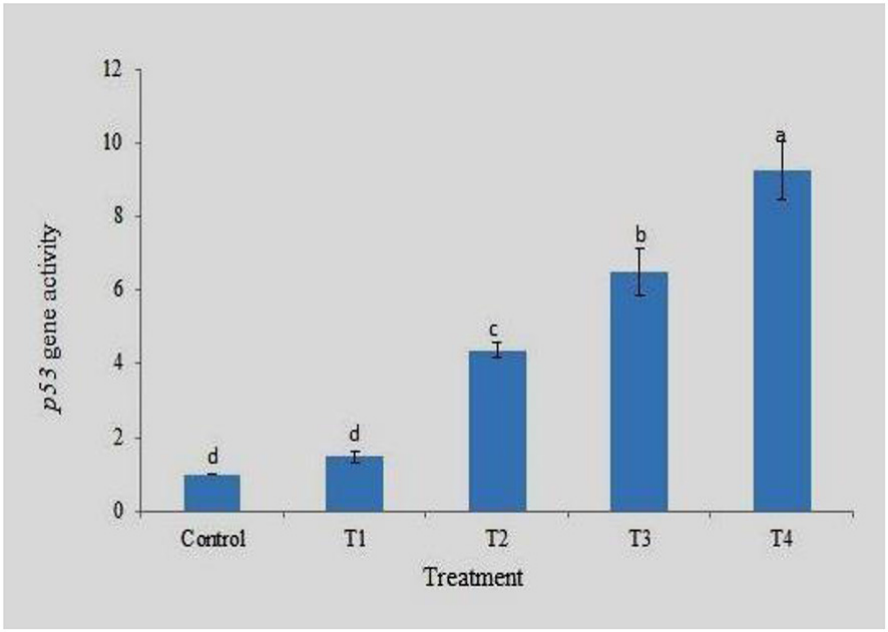
Expression pattern of *p53* gene at different treatment. Data are presented as mean ± SD (*n* = 3). Different superscript letters indicate significant differences among treatments (*p* < 0.05, Tukey's HSD test).

*p53*, also known as a protein-binding protein, is a gene found in the nucleus of every cell in the body that helps regulate normal cell growth and replication. Gene expression analysis clearly showed the highest expression of the *p53* gene in the gills of T4 and the lowest in the control group. The expression level was 42, dependent on the concentration, and an increase in expression was observed with an increase in the drug concentration. Fold change analysis (2^−ΔΔCt^) showed approximately 9.25- and 6.5-times fold change in the expression of *p53* in the gills of T4 and T3, respectively, compared to the control (Figure 9). There was a significant difference in the fold change in gene expression between the different treatments. The results of the MNT and comet assays clearly showed increased genotoxicity with increasing drug concentration. The results clearly show a corresponding increase in *p53* gene expression with an increase in DNA damage.

### *Cyp1a* gene expression

**Figure 10:**
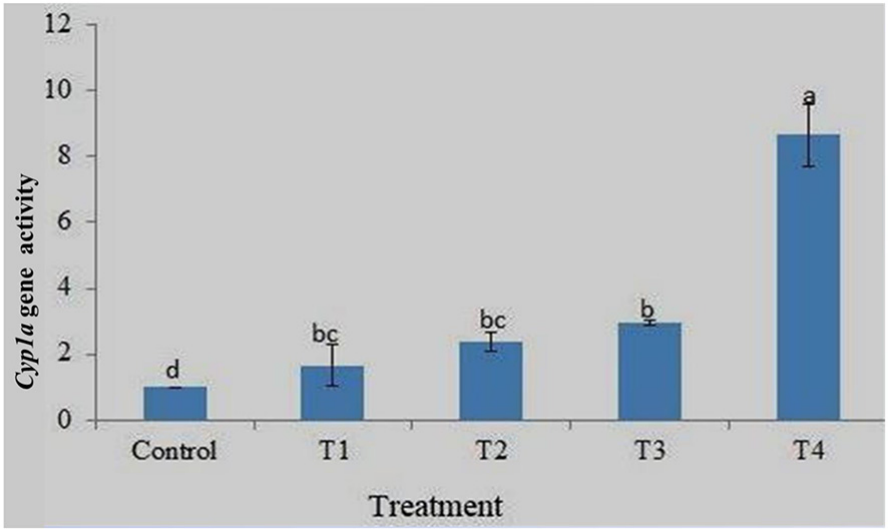
Expression pattern of *Cyp1a* gene at different treatment. Data are presented as mean ± SD (*n* = 3). Different superscript letters on different bars indicate a significant difference (*p* < 0.05, Tukey's HSD test) between different treatments.

Cytochrome P450 monooxygenases stimulate the oxidation and metabolism of large amounts of xenobiotic and endogenous compounds. Cytochrome P450 enzymes are mainly found in liver cells, but they are also present in cells throughout the body. Gene expression analysis clearly showed the highest expression of the *Cyp1a* gene in the gills of T4 and the lowest in the control group. The expression level was concentration-dependent, and an increase in expression was observed with increasing drug concentration. Fold change analysis (2^−ΔΔCt^) showed approximately 8.66- and 2.96-times fold change in the expression of *p53* in the gills of T4 and T3, respectively, compared to the control (Figure 10). There was a significant difference in the fold change in gene expression between the control and all treatments. The results of the MNT and comet assays clearly showed increased genotoxicity with increasing drug concentrations. The results clearly show a corresponding increase in *Cyp1a* gene expression with an increase in DNA damage, but the increases between T1, T2, and T3 were less than those in T4.

### *Bcl-2* gene expression

**Figure 11:**
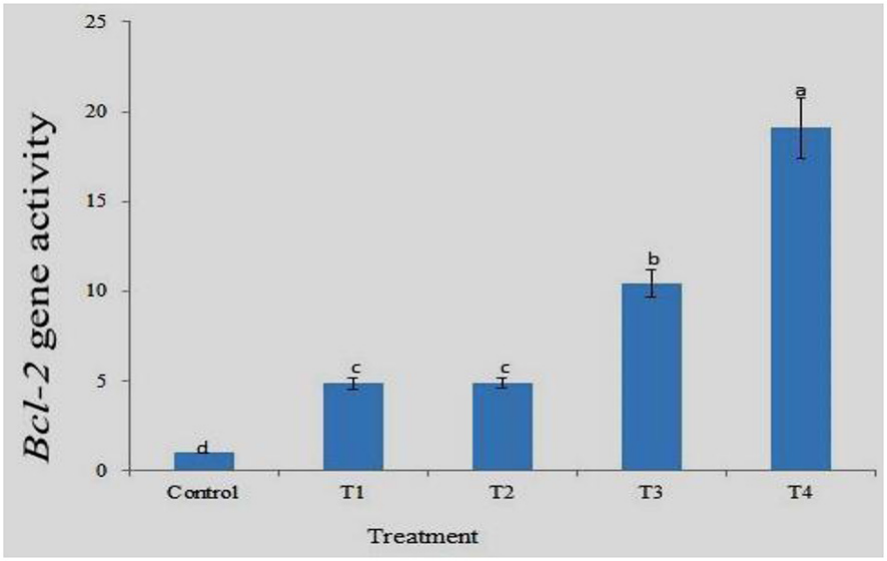
Expression pattern of *Bcl-2* gene at different treatment. Data are presented as mean ± SD (*n* = 3). Different superscript letters on different bars indicate a significant difference (*p* < 0.05, Tukey's HSD test) between different treatments.

*Bcl-2* family proteins are regulators of apoptosis and have other functions. This family of proteins includes inhibitors and inducers of cell death. Together, they control and mediate the process by which mitochondria contribute to cell death, known as the intrinsic pathway of apoptosis. Gene expression analysis clearly showed the highest expression of *Bcl-2* in the gills of the T4 group and the lowest expression in the control group. Expression was concentration-dependent, with higher expression observed at higher drug concentrations. Fold change analysis (2^−ΔΔCt^) showed approximately 19.1- and 10.45-times fold change in the expression of *the Bcl-2* gene in the gills of *T4* and *T3*, respectively, compared to the control (Figure 11). There was a significant difference in fold change of gene expression between the control and all the treatments. The results of the MNT and comet assays clearly show increased genotoxicity with increasing drug concentration. The results clearly show a corresponding increase in *Bcl-2* gene expression with an increase in DNA damage and drug concentration, but the increase between T1 and T2 is less than that between T3 and T4.

## Discussion

A separate *in vivo* experiment was conducted based on the results of the sublethal toxicity study, and four test concentrations (2.5, 5, 7.5, and 10 mg/L) of dichlorvos were selected. The blood samples were analyzed for the presence of micronuclei and comet assay at different time intervals, and the results indicated a concentration-and exposure time-dependent increase in DNA damage, as evident through the increased percentage frequency of micronuclei and tail DNA percentage. A significant increase in micronucleus frequency between T4 and T5 (5.5-fold) was observed after 24 h of exposure, and the trend between T4 and T1 was similar at all sampling points. The lowest MNi frequency in erythrocytes was 0.3519% at 2.5 ppm concentration at 24 h, whereas the highest frequency was 3.4942 ± 0.67645 a at 10 ppm concentration post 96 h. Blood analysis for tail DNA percentage following the comet assay indicated a significantly different degree of DNA damage at different time points at different concentrations, and the damage was again concentration-and exposure time-dependent, similar to MNi frequency. The lowest DNA damage was observed at 24 h at 2.5 ppm, and a gradual non-linear increase in DNA damage in blood cells with the progression of the experiment was observed, reaching a peak after 96 h for all treatments.

The LC_50_ and LC_25_ values post 96 h. exposure were found to be 10.612 and 6.696, respectively. Sişman et al. (13) reported an LC_50_ value of 13 mg/L for DDVP in zebrafish. Our findings are also similar to theirs, and a slight difference of 29 may be due to size variation, water quality parameters, etc. Bhat et al., (14) reported a DDVP LC_50_ value of 16.71 ppm for *Labeo rohita*. The LC_50_ and LC_25_ values vary depending on the fish species, age, size, and hydrological parameters. DDVP is used as an antiparasitic drug; therefore, this LC_25_ value can be used to optimize the dose and duration of treatment for fish for better results without affecting the fish. The result indicates concentration and exposure time-dependent increase in micronucleus frequency as compared to control. A 5.5-fold increase in micronucleus frequency was observed between T1 (2.5 ppm) and T4 (10 ppm) at 24 h. intervals, whereas the increase between T1 and T3 was 3.6-fold. A similar pattern was observed at all time points between the higher and lower DDVP concentrations. Bhatnagar et al., (15) evaluated acute toxicity of chlorpyrifos by exposing *Cirrhinus mrigala* fingerlings and reported significant effects for both concentration and duration of exposure on genotoxic effects. The highest micronucleus was reported on the 14 th day at a chlorpyrifos concentration of 0.08 mg/L. They concluded that chlorpyrifos exerts a genotoxic effect and causes nuclear anomalies in mrigal erythrocytes. Mehra and Chadha (16, 17) evaluated the genotoxic potential of naphthalene 2-sulfonate (2NS) in freshwater fish (*Channa punctatus*). They selected two sublethal concentrations of 2.38 g/15 g body weight (1/4 of LC_50_) and 4.77 g/15 g body weight (1/2 of LC_50_) for acute exposure. They reported dose- and time-dependent DNA damage by comet and micronucleus tests after 60 days. Similarly, dichlorvos and cypermethrin induced significant sublethal toxic effects in *Clarias gariepinus*, including pronounced behavioral abnormalities, increased DNA damage, and progressive ovarian degeneration. Genotoxicity, evidenced by elevated micronuclei and nuclear abnormalities, showed a clear time-dependent increase, indicating cumulative damage with prolonged exposure (18).

Zebrafish exposed to DDVP showed significantly higher DNA damage at higher concentrations compared to lower concentrations and control. DNA damage increased with exposure duration, being lowest at 24 h and highest at 96 h, with a clear concentration-dependent trend observed in both gill tissues and lymphocytes. The toxic effects of dichlorvos were concentration- and exposure time-dependent, and higher concentrations exhibited more genotoxicity, as shown by an increase in % MNi and tail DNA along with increased expression of the gene. The LC_50_ and LC_25_ values of dichlorvos after 96 h in adult zebrafish were 10.612 and 6.696, respectively, and these estimates can be used to design treatment strategies. The blood sample analysis for micronucleus and comet assay at different time intervals indicated a concentration- and exposure time-dependent increase in DNA damage, as evident through increased percentage frequency of micronucleus and tail DNA percentage. A significant increase in micronucleus frequency between T4 and T5 (5.5-fold) was observed after 24 h exposure, and the trend between T4 and T1 was similar at all sampling points. The lowest MNi frequency in erythrocytes was 0.3519% at 2.5 ppm concentration after 24 h, whereas the highest frequency (3.4942 ± 0.67645) was recorded at 10 ppm after 96 h. Similarly, tail DNA percentage showed a gradual non-linear increase with progression of the experiment, reaching a peak after 96 h for all treatments. Similarly, Pandey et al. (19), reported increased DNA damage in *Channa punctatus* using comet assay and RAPD profile changes, indicating DNA breaks in the treatment group. Trivedi et al. (31) reported increased micronuclei formation, DNA damage, and apoptosis under sub-lethal dichlorvos exposure. Ali et al. (20) also reported concentration and exposure time-dependent increases in MNi percentage in *Channa punctatus* exposed to chlorpyrifos. Lower concentrations (less than LC_25_) can be used for treatment of parasites; however, further studies are required.

The expression profiles of the housekeeping genes β-actin, EF1α, and GAPDH were analyzed to determine the most stable internal control for gene expression normalization. Among these, β-actin exhibited the highest stability and was therefore selected as the reference gene for the study. Additionally, melting curve analysis confirmed the absence of non-specific amplification, validating the reliability of the gene expression results. Expression profiling of a few important genes, such as *p53, Cyp1a*, and *Bcl-2*, was performed using real-time PCR. These genes are significantly involved at various levels in the response to DNA damage. Good quality RNA was extracted from gill tissue collected from different treatments after 96 h. Gene expression analysis showed the highest *p53* expression in the gills of T4 and the lowest in the control group. The *p53* gene expression was 9.25fold in 10 ppm and 6.5fold in 2.5 ppm compared to the control at 96 h. The increased expression pattern was a response to DNA damage, as the MNT and comet assays indicated higher damage at 10 ppm. The biological significance of this upregulation lies in the role of *p53* as a cellular defense mechanism against genotoxic stress. The increased expression of *p53* in treated groups indicates activation of DNA damage response pathways, likely triggering cell cycle arrest and promoting DNA repair or apoptosis to prevent the propagation of damaged cells (21). This is consistent with the results of the micronucleus (MNT) and comet assays, which showed increased DNA damage with higher drug concentrations. So, the elevated level of *p53* expression suggest the protective response to counteract genotoxic effects (22). However, excessive activation of *p53* may also indicate severe cellular stress, which could lead to increased apoptosis and tissue damage, particularly in sensitive organs such as gills that are directly exposed to environmental contaminants (23). A similar trend was also observed for *Cyp1a* and *Bcl-2* gene expression, with higher expression at 10 ppm and lowest in the control. The MNT and comet assay indicated an increase in DNA damage with an increase in drug concentration, and a corresponding increase in *p53, Cyp1a*, and *Bcl-2* gene expression was also reported here. Similarly, Williams and Hubberstey (36) analyzed the expression of *p53* and the toxin metabolizing protein, *CYP1A* gene, in liver tissue of bullhead fish collected from clean and contaminated regions of Lake Erie and surrounding water bodies. The results revealed a significantly higher expression of the *p53* gene in fish exposed to contaminated water as an adaptive response to counter the toxic effects. *CYP1A* gene expression was significantly lower, protecting cells by reducing contaminant metabolism, which may produce carcinogens.

Gene expression analysis of the *Cyp1a* gene showed the highest expression in the gill tissue of T4 and the lowest in the control, with a clear concentration-dependent increase across treatments. Fold change analysis (2^−ΔΔCt^) indicated approximately 8.66- and 2.96-fold upregulation in T4 and T3, respectively, compared to the control, with significant differences among treatments. This trend corresponded with the results of micronucleus (MNT) and comet assays, which revealed increased DNA damage at higher drug concentrations. Verbueken et al. (24) evaluated cytochrome P450 activity and related detoxification genes in zebrafish embryos and larvae using specific substrates and qPCR analysis. Their results showed that these proteins play a minimal or undetectable role in xenobiotic metabolism before 72 h post-fertilization. Similarly, Mohamed et al. (25) reported that exposure to hexavalent chromium (Cr VI) in *Oreochromis niloticus* led to increased caspase-3 expression, decreased *Bcl-2*, reduced growth, and downregulation of *Cyp1a* and GST genes, along with tissue damage and inflammatory changes. The biological significance of *Cyp1a* upregulation lies in its role as a key component of the xenobiotic detoxification system. These enzymes catalyze the oxidation and metabolism of toxic compounds, particularly in gill tissues that are directly exposed to environmental contaminants. The increased expression in treated groups reflects an adaptive response to enhance detoxification capacity under chemical stress. However, *Cyp1a* enzymes can also bioactivate xenobiotics into reactive intermediates, leading to oxidative stress and DNA damage. This explains the observed correlation between increased gene expression and genotoxicity. The higher induction in T4 suggests that at elevated concentrations, detoxification mechanisms may become overwhelmed, contributing to cellular damage. Thus, *Cyp1a* expression serves as an important biomarker of both adaptive response and toxicity.

The *Bcl-2* family proteins are crucial regulators of apoptosis, acting as both inhibitors and promoters of cell death, and play a central role in the mitochondrial (intrinsic) pathway. In the present study, gene expression analysis showed the highest expression of the *Bcl-2* gene in the gill tissue of T4 and the lowest in the control group, with a clear concentration-dependent increase across treatments. Fold change analysis (2^−ΔΔCt^) revealed approximately 19.1- and 10.45-fold upregulation in T4 and T3, respectively, compared to the control, with significant differences among all groups. This pattern was consistent with micronucleus (MNT) and comet assay results, which indicated increased DNA damage with rising drug concentrations. The biological significance of *Bcl-2* upregulation lies in its anti-apoptotic function. Increased expression suggests an adaptive response to genotoxic stress, where cells attempt to inhibit apoptosis and maintain survival despite DNA damage (26). This is particularly important in gill tissues, which are directly exposed to environmental contaminants. The relatively lower expression in T1 and T2, compared to T3 and T4, indicates that this protective mechanism becomes more active under higher stress conditions. Similarly, Zheng et al. (27) reported altered expression of *Bcl-2* and related genes in zebrafish exposed to PBDE (BDE-47), indicating stress-induced molecular changes. Cobbina et al. (28) also observed significant upregulation of *Bcl-2* in fish exposed to heavy metals, suggesting a protective response against toxicants. However, Cao et al. (29) demonstrated that prolonged exposure to fluoride in *Cyprinus carpio* led to oxidative stress and apoptosis, highlighting that sustained stress can overwhelm cellular defense mechanisms.

Our results suggests that dichlorvos (DDVP) causes DNA damage in zebrafish, and this effect increases with higher doses and longer exposure time with activation of key marker genes such as stress (*p53*), detoxification (*Cyp1a*), and apoptotic (*Bcl-2*) pathways, even at sublethal concentrations. This indicates that while DDVP may be effective as an antiparasitic agent, but due to its narrow safety range requires careful dose optimization in aquaculture. Therefore, further studies should focus on parasite-specific efficacy and safer therapeutic dose ranges to support sustainable and environmentally responsible aquaculture practices.

## Conclusion

Disease management is currently of primary importance for the sustainable growth of aquaculture and requires various preventive and post-outbreak management strategies. Parasitic diseases, although not as serious as other infectious diseases, still cause extensive damage to aquaculture, mainly affecting the growth and reproductive performance of fish. The strategies adopted by farmers is mainly dominated by the application of antiparasitic drugs/chemicals. These chemicals reach aquatic ecosystem along with agricultural runoff water and affect many non-target organisms, such as fish, gastropods, and crustaceans. The toxic effects of antiparasitic chemicals on the genetic material (genotoxicity) of the host organism are a major concern and need to be evaluated to understand their safety levels. The present study demonstrates that dichlorvos (DDVP) induces significant genotoxic effects in zebrafish, DNA damage, and altered gene expression patterns. The estimated LC_50_ (10.612 ppm) and LC_25_ (6.696 ppm) values after 96 h indicate the toxic potential of DDVP even at sublethal concentrations. The *in vivo* exposure to selected concentrations (2.5–10 mg/L) further confirmed dose-dependent genotoxicity. These findings highlight the ecological risk posed by dichlorvos in aquatic environments and emphasize the need for species-specific toxicity assessments and cautious application of such pesticides.

The expression profiling revealed that the *p53* gene expression was 9.25fold in 10 ppm and 6.5fold in 2.5 ppm compared to control at 96 h. A similar trend was also observed with *Cyp1a* and *Bcl-2* gene expression, with higher expression at 10 ppm and lowest in control. The MNT and comet assay indicates an increase in DNA damage with the increase in drug concentration, and a corresponding increase in *p53, Cyp1a* and *Bcl-2* gene expression. It can be said that DDVP has caused diverse effects, like DNA damage, stress and genotoxicity effects in fish at a chronic level. The toxic effects of dichlorvos on zebrafish were concentration and exposure time-dependent, and higher concentrations exhibit more genotoxicity, as shown by an increase in % Mni and tail DNA along with increased expression of the gene. The lower concentration (less than LC_25_) can be used for treatment against the parasites. Further studies are required to check the efficacy of DDVP against different parasites to formulate more specific treatment strategies. The effects of the drug on fish reproductive performance need to be analyzed.

## Data Availability

The original contributions presented in this study are included in the article. Further inquiries can be directed to the corresponding author.
